# Fellow-Workers and the Roll of Honour

**Published:** 1915-02-20

**Authors:** 


### ROUND THE WORLD.

[The Editor invites Early Information and Contributions Marked "Fellow-Workers.'

### Fellow-Workers and the Roll of Honour.

The radiographic department of the King George Hospital will be administered by three specialists whose names are well known as those of responsible investigators of *x*-ray phenomena, and as having extensive experience of medical and surgical radiography. They are Dr. W. Ironside Bruce, Dr. Stanley Melville, and Dr. G. Harrison Orton. A department of this kind is, of course, an expensive necessity, and it is anticipated that its equipment at the institution named will cost the best part of a thousand pounds.

Dr. William Ironside Bruce, M.D., Ch.B. Aberd., whose keen work in radiography has brought him a great reputation early in his career, is physician to the *x*-ray and electrical departments at Charing Cross Hospital and honorary radiographer to the Hospital for Sick Children at Great Ormond Street; he also supervises the similar departments at the Hospital of SS. John and Elizabeth in St. John's Wood. Dr. Ironside Bruce served as civil surgeon with the South African Field Force during the war, and is the author of a "System of Radiography with Atlas of the Normal," as well as of important articles on "*X*-ray Examination and Treatment" in Choyce's "System of Surgery" and Latham and English's "System of Treatment."

Dr. Stanley Melville, M.D. Brux., is medical officer in charge of the *x*-ray department at the Hospital for Diseases of the Chest, Brompton, and a former student of University College, London, and Liverpool, who qualified by taking the diploma of the apothecary's department in 1891. Dr. Melville has done, and continues to carry out, a great deal of hospital work, for in addition to his Brompton appointment he is radiographer to the Victoria Hospital for Children at Chelsea, to the National Orthopædic Hospital, to the Italian Hospital, and to the Croydon General Hospital; whilst formerly he was assistant radiographer at Charing Cross Hospital and to the Hospital for Sick Children. He has published many interesting observations on the diagnosis of consumption by *x*-rays. Dr. Melville is also a barrister-at-law.

Dr. George Harrison Orton, M.D., is a Cambridge and "Bart.'s" man, who now occupies the responsible positions of medical officer in charge of the *x*-ray department at St. Mary's Hospital and at the Paddington Green Children's Hospital. Qualifying M.B., B.C. Cantab., M.R.C.S., L.R.C.P. Lond., in 1901, he soon devoted himself to *x*-ray treatment, and became chief assistant to the electrical department at St. Bartholomew's Hospital, as well as radiographer to the Royal Free Hospital and to the Cheyne Hospital for Sick Children at Chelsea, appointments which he has since relinquished. Dr. Harrison Orton is the author (with Dr. Hugh Walsham) of a book on "Röntgen Rays in the Diagnosis of Diseases of the Chest," as well as of important observations on the "*X*-ray Diagnosis of Renal and Urethral Calculi," and of other communications on his special subject.

The New Zealanders fighting in Egypt are unfortunate in having lost the services of a capable medical officer, Captain James Alexander Terras Bell, whose death has just been reported. Captain Bell was an Edinburgh man, and qualified at his University in 1895, subsequently becoming an M.D. Edin. with honours. After qualification he increased his clinical experience at the Brompton Hospital for Consumption, and also at the Cardiff Infirmary, where he served as assistant house physician. Then going to South Africa, he fulfilled the duties of assistant resident surgeon at the Somerset Hospital at Cape Town and plague medical officer at Port Elizabeth. Whilst in South Africa he was interested in the Cape Medical Staff Corps, and obtained a commission in that body as surgeon-captain. Captain Bell was apparently of a roving disposition, for after spending some time at the Cape he went to New Zealand, and started practice in Christchurch, where he became honorary physician to the hospital. At the outbreak of war he felt the call to foreign service, and came back to Europe with the New Zealand contingent.

Mr. Herbert Furnivall Waterhouse, M.D. Edin., F.R.C.S. Eng., who has been appointed surgeon to the King George Hospital, served as resident surgeon to the Royal Infirmaries of Edinburgh and Glasgow, and then, coming up to London, became elected a member of the staff of Charing Cross Hospital, where he is now surgeon and lecturer on surgery, and was formerly dean, lecturer in anatomy, and lecturer on operative surgery. Mr. Waterhouse is also senior surgeon to the Victoria Hospital for Children, consulting surgeon to the Kensington Children's Hospital, and is well known in London as an operator. At the present time he examines in surgery for the Universities of London and Manchester.

Mr. Herbert Sherwell Clogg, who is a colleague of the last-named at the new institution, studied medicine at Cardiff and Charing Cross Hospital. He qualified by taking the diploma of the Conjoint Board, and subsequently became an F.R.C.S. Eng.; at the same time, pursuing the London University course, he took the exhibition and gold medal in anatomy at the Intermediate M.B., and the gold medal at the M.S. Lond. He also is a member of the staff of Charing Cross Hospital, where he is surgeon to out-patients. Mr. Clogg, who has written largely on abdominal conditions, also holds the appointments of senior surgeon to the Evelina Hospital for Sick Children and lecturer on operative surgery at Charing Cross Hospital.

The late Lieutenant William Ebenezer Maitland was a medical man who entered the combatant ranks on the outbreak of war. A student of Glasgow University, where he became an M.B., Ch.B. two years ago, he was fulfilling the duties of house physician at the Glasgow Royal Infirmary when the international crisis occurred last August. Being successful in obtaining a commission as second lieutenant in the Seaforth Highlanders (3rd Battalion), Mr. Maitland gave up his medical post and was soon sent out to the Front.

Lieutenant Geoffrey Hadfield, who has been given the responsible post of pathologist in the British military hospital at Rouen, has lately been working as medical registrar and assistant pathologist to the Seamen's (*Dreadnought*) Hospital at Greenwich. A "Bart 's" man, he qualified M.B., B.S. Lond. in 1911, and two years after carried off the gold medal at the M.D. examination. Subsequently he held the appointments of house physician at St. Bartholomew's Hospital, and senior resident and senior house surgeon to the Metropolitan Hospital.

